# Formation of yttria-stabilized zirconia nanotubes by atomic layer deposition toward efficient solid electrolytes

**DOI:** 10.1186/s40580-017-0127-9

**Published:** 2017-12-05

**Authors:** Eunsoo Kim, Hyunchul Kim, Changdeuck Bae, Daehee Lee, Jooho Moon, Joosun Kim, Hyunjung Shin

**Affiliations:** 1 Department of Energy Science, Sungkyunkwan University, Suwon, 440-746 South Korea; 2 Integrated Energy Center for Fostering Global Creative Researcher (BK 21 plus), Sungkyunkwan University, Suwon, 440-746 South Korea; 3 Department of Materials Science and Engineering, Yonsei University, Seoul, 120-749 South Korea; 4 High-Temperature Energy Materials Research Center, Korea Institute of Science and Technology, Seoul, 136-791 South Korea

**Keywords:** Atomic layer deposition, YSZ nanotubes, Solid oxide electrolytes

## Abstract

We describe a fabrication strategy for preparing yttria-stabilized zirconia nanotube (YSZ-NT) arrays embedded in porous alumina membranes by means of template-directed atomic layer deposition (ALD) technique. The individual YSZ-NTs have a high aspect-ratio of well over 120, about ~ 110 nm in diameter, and ~ 14 µm in length. Interfacing the tube arrays with porous Pt was also introduced on the basis of partial etching technique in order to construct Pt/YSZ-NTs/Pt membrane electrode assembly (MEA) structures. The resulting YSZ-NTs MEAs show a 7 mm in diameter with a roughness factor of ~ 2. Area specific resistance was measured up to 1.84 Ω cm^2^ at 400 °C using H_2_ as fuel.

## Introduction

Owing to high diffusivity for oxygen ions [1], exceptional resistance against mechanical and thermal stress [2], electrical insulation [3] and high biocompatibility [4], zirconia-based materials have many industrial applications. Notable examples are solid oxide fuel cells [5–7], catalysts [8], thermal barrier coatings [9], jet engines [10], alternative gate-oxides in microelectronics [11, 12], and implantable biomaterials for a hip joint [13]. Bulk ZrO_2_ has three well-known polymorphisms at normal atmospheric pressure [14]. The monoclinic baddeleyite structure (*m*-ZrO_2_) is thermodynamically stable under ambient conditions, where Zr atoms are in a distorted sevenfold coordination, and O atoms have four- or three-fold coordination. *m*-ZrO_2_ transforms reversibly to the tetragonally distorted fluorite structure (*t*-ZrO_2_) above ~ 1175 °C, with Zr in an eightfold coordination. This phase transformation is known to be accompanied by a substantial volume change of ~ 5 vol.%. Cubic fluorite structured ZrO_2_ (*c*-ZrO_2_) is the most stable one and is stabilized upon ~ 2370 °C. Stabilization of *t*-ZrO_2_ and *c*-ZrO_2_, which is required to be technologically viable over *m*-ZrO_2_, is of significant importance in many applications. In order to stabilize the cubic structure even down to room temperatures, adding aliovalent oxides such as CaO, Y_2_O_3_ and Gd_2_O_3_ has been suggested [15].

In principle, formation of the oxygen vacancies plays a major role in applications using zirconia-based ceramics. The vacancy diffusivity depends not only on the phase itself [15], but also on the strain [16], space-charge [17, 18], and defect localization [19] effects. Therefore, microstructures such as grain boundaries and defect densities are essential in determining the materials properties [20]. For example, thin film/layer electrolytes of doped-ZrO_2_ exhibited enhanced performance at lower operation temperatures in application for solid oxide fuel cells (SOFCs) [21, 22]. Currently, YSZ is the most common solid-electrolyte materials for SOFCs.

To accomplish adequate ionic conductivity in conventional ZrO_2_-based electrolytes, SOFCs generally require an operating temperature above 850 °C. Such high operating temperatures require severe demands on the materials used to satisfy chemical as well as thermal stability [23]. Quite long start-up and shutdown time is also limited to many applications, such as portable power and transportations [24]. There are considerable interests in bringing the operating temperature down to intermediate range (600–800 °C) and even to lower (< 500 °C) temperature. At even lower temperature, system costs can significantly reduce due to wider range of materials used for components. On the other hand, low operating temperature could cause reducing significantly the ionic conductivity of the solid electrolyte and thus leads to higher ohmic losses. Ohmic loss is governed by the ionic conductivity and the layer thickness of electrolytes. The first approach is to introduce novel electrolytes with higher ionic conductivity at lower temperatures such as La_0.9_Sr_0.1_Ga_0.8_Mg_0.2_O_3-δ_ (LSGM) [25], and Sm_0.075_Nd_0.075_Ce_0.85_O_2-δ_ (SNDC) [26]. These have generally shown lower chemical stability than that observed in YSZ electrolytes. The second approach is to extremely reduce the layer thickness of electrolyte less than 100 nm, i.e. to decrease the diffusion path of oxygen ions [10]. Typical MEA structure for thin-film SOFCs is a planar thin-film membrane with two porous electrodes separated by a thin and at the same time dense, air-tight, oxygen-ion conducting electrolyte. Micrometer-thick MEAs have been fabricated using micro-electro-mechanical systems (MEMS) processing based on silicon wafers using chemical etching as mechanical support for free-standing ultrathin MEA [27–32]. Several substrates have been used as alternate support materials for MEA, including nickel foil [33], porous nickel cermet [34], glass-ceramic [35], and AAO substrates [36–39]. Moreover, three-dimensional (3D) nanostructured MEAs also increased the cell performance due to the increased active surface area. Chao *et al*. reported a corrugated MEA by nanosphere lithography which active membrane area was enhanced to 1.6–twofold [40, 41]. Su et al. also reported a cup-shape MEA at micrometer scale which roughness factor of active surface area was increased to ~ 5 [42]. In order to achieve extremely large roughness factors, a natural occurrence is to employ the nanotubular geometry with high aspect ratio. However, the resulting power density was rather disappointed down to only 1 μW cm^−2^ [43], calling for the emergent design using zirconia-based nanotubes with high aspect ratio as solid electrolytes.

To fabricate the 3D nanostructured MEAs, ALD is one of the most ideal deposition technique of choice. ALD is a gas phase thin film deposition based on alternate, self-limiting surface reaction of precursors [44]. ALD allows for fabricating high aspect-ratio and complex surface structures employing templates such as AAO [45–48], Opals [49], and aerogel structures [50]. And also, nanoscale laminated films can be grown by alternative depositions at each atomic layer with desired ratio of the number of deposition cycles [51]. In this paper, we studied on the formation of YSZ NTs by template-directed ALD. By controlling the atomic layer depositing ratio of ZrO_2_ and Y_2_O_3_, we were able to achieve the cubic phase YSZ-NTs with high aspect-ratio up to ~ 110. To expose the large active surface area of YSZ-NTs, partial etching procedures were developed. The resulting free-standing YSZ-NT-based MEA was prepared having porous Pt electrodes at the both sides with an active area of 7 mm in diameter. The preliminary results exhibit promising resistance values under H_2_ ambient at 400 °C.

## Experimental

AAO templates were fabricated by a two-step anodization method base on aluminum (99.999%, Goodfellow, UK) [52–54]. The aluminum foils were electropolished with a mixture of HClO_4_/EtOH (1:3 vol.%) at 18 V for 4 min. The first anodization was performed in 1 wt.% H_3_PO_4_ solution at 0.5 °C under applied DC voltage of 195 V for 16 h. Then, the alumina layers were removed wet-chemically in a mixture of 6 wt.% H_2_CrO_4_ and 1.8 wt.% H_3_PO_4_ at 45 °C for 24 h. The second anodization was done under the identical conditions to the first one for a desired time. Approximately, 11 μm-thick templates were prepared upon ~ 4 h. The pore widening was proceeded in 10 wt.% H_3_PO_4_ solutions at 45 °C for 30 min, when increased for pore diameter up to ~ 200 nm. Separation of AAO template from the aluminum foil could be accomplished by wet-chemically etching aluminum with a mixture of 3.4 wt.% CuCl_2_ in water and 37% HCl solution.

ZrO_2_ and YSZ were grown on the AAO templates using a commercial ALD reactor (TFS-200, Beneq, Finland) at 200 °C. Tetrakis(ethylmethylamino)zirconium [TEMAZr] (UP Chem., Korea) and Tris(methylcyclopentadienyl)yttrium [(MeCp)_3_Y] (Strem Chem., USA) were used as metal containing reactants preheated at 80 and 130 °C, respectively. Deionized water was used as oxygen source and delivered at room temperature. Dried N_2_ was used as purge/carrier gas. Both ZrO_2_ and YSZ thin films depositions were used in expose-mode protocol, where a full ALD cycle is consisting of 10 s pulse, 50 s waiting, and 60 s purging. For the deposition of cubic phase YSZ films, 7 ALD cycles of ZrO_2_ layer and 1 ALD cycle of Y_2_O_3_ layer were repeatedly deposited for desired thickness [16]. Porous Pt thin films were deposited on both cathode and anode by DC sputtering (Cressington 308R, Cressington, UK) at room temperature in 10 Pa Ar ambient[15].

Physical dimensions of the resulting tubes and MEAs were inspected by field-emission scanning electron microscopy (FE-SEM, JEOL JSM-7000F, Japan & Carl Zeiss AG SIGMA, Germany). The structures of as-grown and post-annealed nanotubes were investigated by X-ray diffraction (XRD, Rigaku Ultima IV, Japan) and high-resolution transmission electron microscopy (HRTEM, JEOL JEM-4010, Japan). And also, specific crystal structure of nanotubes was obtained using a synchrotron radiation source at beam line 5A with the wavelength 0.7653 Å at Pohang Light Source using Mar 345-image plate. For converting image to the 2D diffraction pattern, FIT2D program was used [55]. The 2*θ* of XRD pattern was recalculated to the corresponding angles of λ = 1.54 Å (Cu-Kα radiation). The electrochemical impedance spectroscopy was measured to determine the polarization resistance of the cells using potentiostat and frequency analyser (1252, Solartron, UK) in the frequency range of 100 kHz–0.1 Hz with AC amplitude of 20 mV.

## Results and discussion

**Fig. 1:**
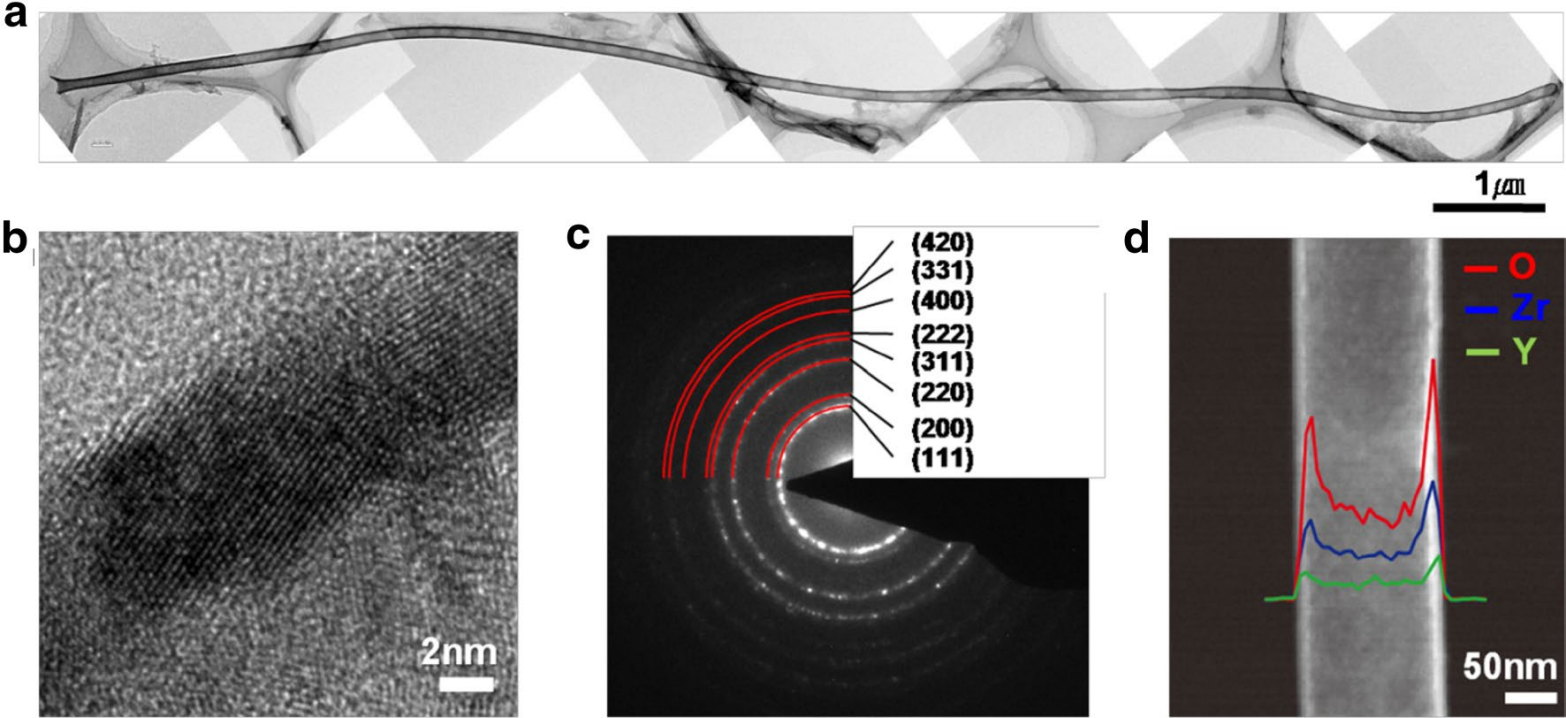
TEM micrographs of YSZ NTs grown by repeating a full ALD cycle consisting of 1 yttria and 7 zirconia sub-cycles. **a** Low-magnification image, exhibiting a high aspect-ratio of over 120, 110 nm in diameter, and 14 μm in length. Note that one distal end is open. **b** The lattice image, showing that the YSZ-NTs are formed with well-crystallized nanometer sized grains of less than 10 nm in diameter, as grown. **c** The selected electron area diffraction (SEAD) patterns are consistent with cubic phase of zirconia (JCPDS #30-1468). **d** EDS elemental line profiles across a YSZ NT also confirmed Y-doped ZrO_2_

Doping of aliovalent oxides stabilizes the cubic phase of ZrO_2_ [56], and the substitutional cations (Ca^2+^, Mg^2+^, Y^3+^) will generate oxygen vacancies for charge compensation which makes a fast ionic conductor. The amount of Y_2_O_3_ in the ZrO_2_ matrix could be controlled by varying the ratio of number of cyclic ALD process of ZrO_2_ and Y_2_O_3_. Shim et al. reported that ZrO_2_:Y_2_O_3_ = 7:1 ratio is estimated to be 7–8 mol% of Y_2_O_3_ in the ZrO_2_ matrix [16], as an optimum for high ionic conductivity [57]. Our results also exhibited similar doping behaviors (see Fig. 1d).

Figure 1a shows a mosaic of TEM images of YSZ-NTs by the template-directed ALD method and subsequent wet-chemical etching of the templates. The resulting YSZ-NTs have no-cracks and pinhole-free with a high aspect ratio over 120, ~ 110 nm in diameter, and ~ 14 μm in length. Since YSZ-NTs function as electrolyte for the oxygen transport as well as anti-fuel-crossover layers, even the small pinholes and cracks could result in severe damage to the performance and failure of cells. HR-TEM image and selected electron area diffraction (SAED) patterns confirmed that YSZ-NTs were polycrystalline with the grain size of sub 10 nm in the cubic polymorphism (Fig. 1b, c). Figure 1d shows the energy-dispersive X-ray spectroscopy (EDS) elemental line profile taken from the YSZ-NT. The green line indicated for yttrium which confirmed the presence of Y_2_O_3_ as dopant in the ZrO_2_ matrix (to be 6–8 mol%).

**Fig. 2:**
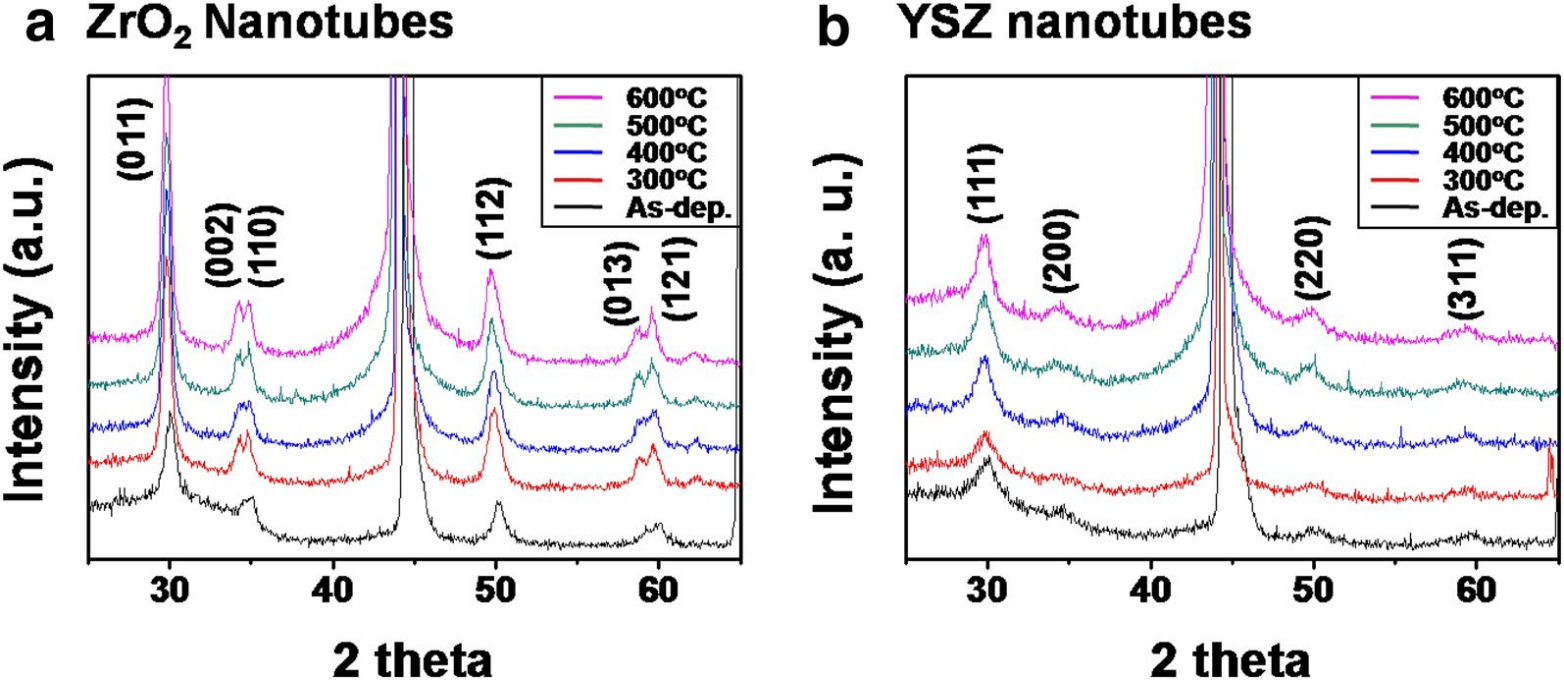
XRD patterns with and without yttira doping at various annealing conditions. **a** ZrO_2_ NTs exhibited tetragonal phase (JCPDS #50-1089), and **b** YSZ NTs, cubic phase (JCPDS #30-1468)

**Fig. 3:**
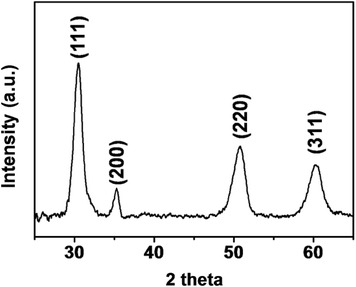
XRD patterns of YSZ-NT arrays from beam line 5 A with the wavelength 0.7653 Å using Mar 345-image plate at Pohang Light Source (South Korea). For converting image to the 2D diffraction pattern, FIT2D program was used. The 2*θ* of XRD pattern was recalculated to the corresponding angles of λ = 1.54 Å (Cu-Kα radiation)

With and without applying the Y_2_O_3_ sub-cycles, the resulting structures were analyzed by XRD upon thermal annealing at different temperatures up to 600 °C. The observed XRD patterns showed the noticeable difference at around 35  each other. The ZrO_2_ NTs without doping stabilized in the tetragonal phase, which is in good agreement with our previous works, due to the developments of nanosized grains [58, 59]. An XRD pattern for the tetragonal phase of ZrO_2_ NTs is shown in Fig. 2a. The XRD pattern clearly observed the peak splitting at around 35° and 60  of 2θ which are indicated (002) (110) and (013) (121), respectively. Remarkably, the structures of the resulting YSZ-NTs differ from those of ZrO_2_ NTs although the tetragonal and cubic zirconia results in very similar XRD patterns. The cubic phase of YSZ NTs exhibited the only single peaks that assigned to (200) and (311) at 34.9° and 59.6  of 2θ, respectively. Note that the measurement conditions were identical for all the samples in Fig. 2. To confirm the exact structure whether or not our YSZ-NTs have the cubic phase as-grown, a synchrotron source was used to obtain diffraction patterns further. The high intensity and collimation of synchrotron radiation allowed for high resolution studies and also results very close to Bragg reflections. As shown in Fig. 3, the peaks at 30.1°, 34.9°, 50.2°, and 59.6  of 2θ unambiguously assigned the cubic phase, corresponding to the (111), (200), (220) and (311) planes, respectively.

**Fig. 4:**
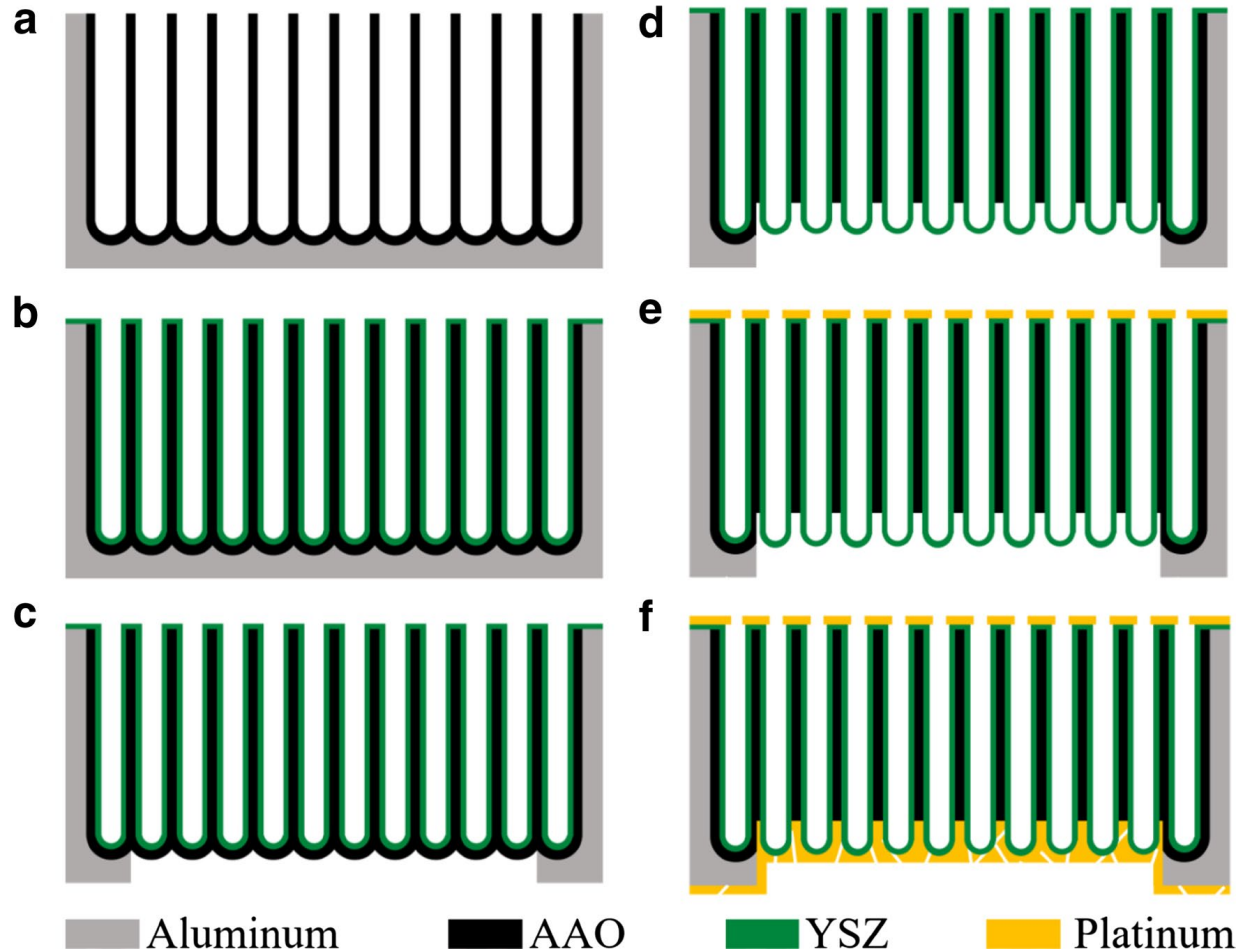
Fabrication steps for the YSZ-NT array-based MEA. **a** Preparation of AAO templates. **b** Deposit the YSZ thin films into the alumina membrane by ALD. **c** Remove the back-side Al by wet chemical etching. **d** Partially etching the alumina membrane in order to expose the tube ends. **e** Sputter porous Pt electrodes onto the top side membrane. **f** Sputter another porous Pt electrodes on the bottom side

Once the target materials and structures were achieved, we developed a fabrication strategy for preparing YSZ-NT-based MEA structures as illustrated in Fig. 4. First, YSZ thin films were deposited into the AAO template by ALD as described above. To expose the close ends of YSZ-NTs surface, the Al and AAO layers were partially removed by wet-chemical etching in the mixed solution of CuCl_2_ + HCl and 10 wt.% H_3_PO_4_. Finally, porous Pt electrodes were deposited on both the top and bottom sides of free-standing YSZ NTs with AAO templates. Figure 4 show the photographs of free-standing MEAs with Al foil and AAO template as mechanical supports. On the top side, the Pt electrode was formed inside the alumina membranes to prevent electrical contact with the aluminum ring. From the bottom side, we clearly verified that the dimension of the free-standing MEA area is 7 mm in diameter. The SEM images show that porous Pt electrode continuously covered the both sides of YSZ-NTs. On the bottom side, the end-closed YSZ-NTs were exposed from the template by partial etching, and the height of exposed YSZ-NTs approximately 300 nm. This nanotubular array structures offered a geometrical surface area ~ 2 times larger than the projected area of the planar structure.

**Fig. 5:**
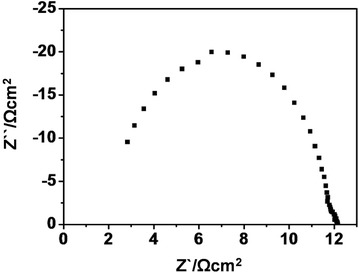
Nyquist plot of the impedance spectra of the YSZ-NT array-based MEA measured under H_2_ ambient at 400 °C

**Fig. 6:**
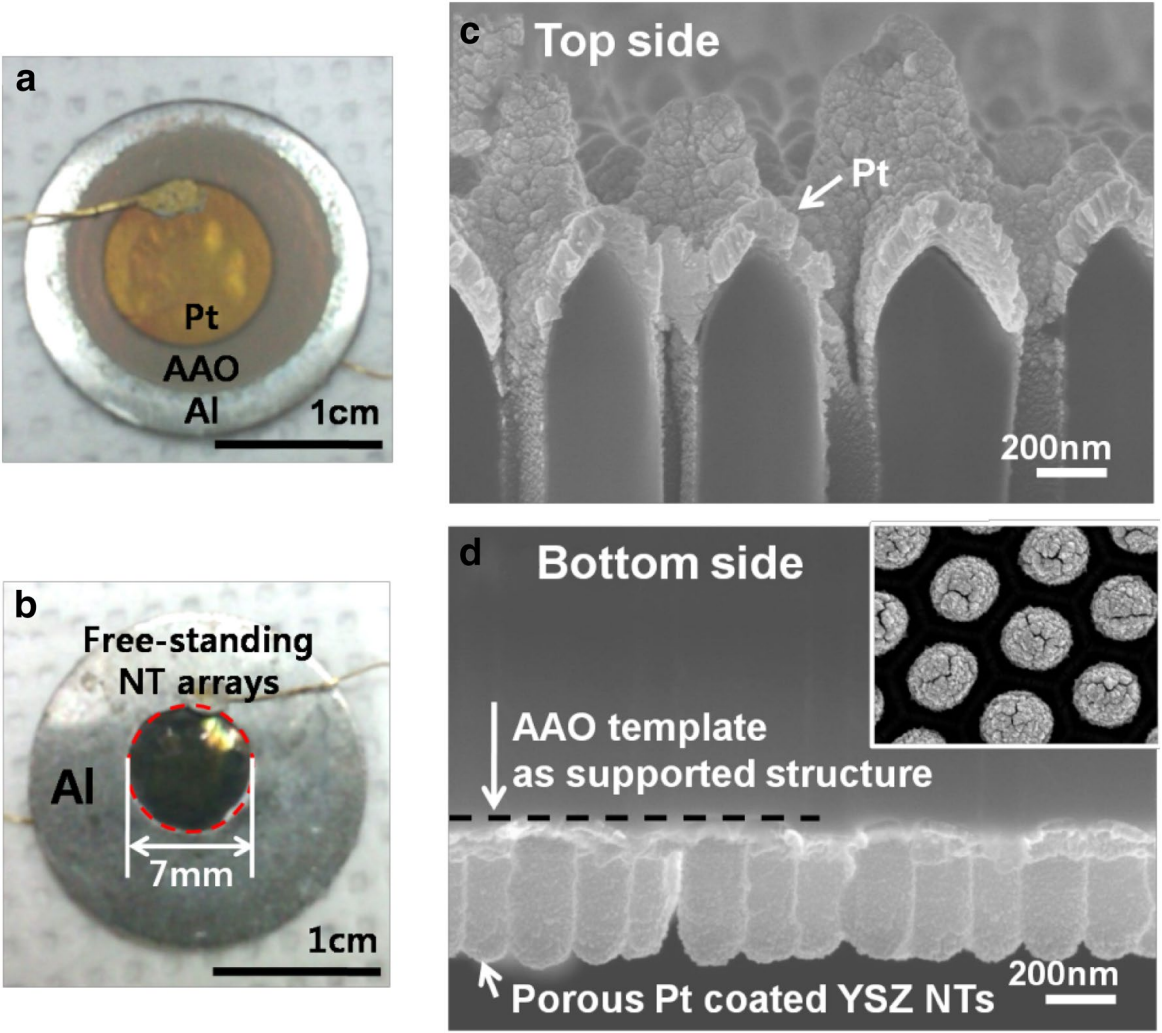
**a**, **b** Photographs of free-standing YSZ-NT-based MEA with an active area of 7 mm in diameter. **a** Golden color shows porous Pt electrode on the YSZ NTs; gray color, YSZ NTs on AAO template; metal color, Al disk. **b** The corresponding back-side image, exposing the free-standing YSZ-NTs (dark green). **c** Cross-sectional SEM image of the top-side MEA, displaying porous Pt electrodes with ~ 80 nm thickness on the YSZ-NT membranes by sputtering. **d** SEM micrographs of porous Pt electrode coated on the exposed tubes in the partially etched alumina template. Inset, the plane view

The charge transfer resistance at moderate temperatures pertains to application in solid oxide fuel cells. We monitored the resulting resistance of our free-standing YSZ-NT-based MEA structures at 400 °C using H_2_ as fuel. Figure 5 shows the corresponding impedance spectra. The high-frequency intercepts, corresponding to the ohmic resistance due to ionic transport through the electrolyte [60], were approximately 1.84 Ω cm^2^. With the porous electrodes, the ohmic resistance was higher than similar roughness factor of nanostructued MEAs [25], because of the distance between top and bottom electrode over 10 μm and hollow structure of the NTs. Toward the higher power density, the pores inside the YSZ-NTs should be completely filled with porous electrodes to shorten the electrode-electrode distance which in turn reduces the resistance of the cell further (Fig. 6).

## Conclusion

In summary, we studied on the formation of YSZ-NTs with high aspect ratio up to ~ 110 by template-directed ALD method. The crystal structures of tetragonal phase ZrO-_2_ NTs and cubic Phase YSZ NTs were demonstrated using both conventional XRD and high-resolution synchrotron radiation diffraction, complementarily. And also, the YSZ NTs based MEAs with AAO template as mechanical support structures were introduced by wet chemical etching technique. Using this fabrication process, the total area of the free-standing MEA and the roughness factor have achieved up to 7 mm in diameter and approximately ~ 2 times, respectively. The present study will open a new venue for realizing the micro-SOFCs with ultra-high efficiency and low-temperature operation capability.
